# Interaction between PAF and human platelet membranes: a fluorescence study

**DOI:** 10.1155/S0962935192000218

**Published:** 1992

**Authors:** Ahmad Kantar, Pier Luigi Giorgi, Giovanna Curatola, Rosamaria Fiorini

**Affiliations:** 1 Department of Pediatrics University of Ancona via Corridoni 11 Ancona l-60123 Italy; 2 Department of Biochemistry University of Ancona via Corridoni 11 Ancona l-60123 Italy

## Abstract

The interaction between PAF and human platelet membranes was investigated by measuring the steadystate fluorescence anisotropy and fluorescence decay of 1 (4-trimethylammoniumphenyl)-6-phenyl-1,3,5-hexatriene (TMA-DPH) incorporated in platelet plasma membranes. PAF induced a time-limited and significant increase of the lipid order in the exterior part of the membrane and a decrease in membrane heterogeneity. These changes were blocked in the presence of the PAF antagonists, L-659,989 and 1-O-hexadecyl-2-acetyl-sn-glycero-3-phospho(N,N,N-trimethyl)hexanolamine.H_2_O. These results indicate that the observed changes in the physico-chemical properties of the membrane are attributed to the PAF–receptor interaction and signal transduction.


Mediators of Inflammation, 1, 127–131 (1992)

### Interaction between PAF and human platelet membranes: a fluorescence study

**Ahmad Kantar,[1,CA] Pier Luigi Giorgi,[1] Giovanna Curatola[2] and Rosamaria Fiorini[2]**

Departments of Pediatrics[1] and Biochemistry[2], University of Ancona, via Corridoni 11, I-60123 Ancona, Italy

[CA] Corresponding Author

**THE interaction between PAF and human platelet membranes was investigated by measuring the steady-state fluorescence anisotropy and fluorescence decay of 1-(4-trimethylammoniumphenyl)-6-phenyl-1,3,5-hexatriene (TMA-DPH) incorporated in platelet plasma membranes. PAF induced a time-limited and significant increase of the lipid order in the exterior part of the membrane and a decrease in membrane heterogeneity. These changes were blocked in the presence of the PAF antagonists, L-659,989 and 1-*O*-hexadecyl-2-acetyl-*sn*-glycero-3-phospho(*N,N,N*-trimethyl)hexanolamine.$H_2O$. These results indicate that the observed changes in the physico-chemical properties of the membrane are attributed to the PAF–receptor interaction and signal transduction.**

**Key words:** Fluorescence, Membrane heterogeneity, PAF, PAF antagonists, Platelet membrane, TMA-DPH

### Introduction

Platelet-activating factor (PAF, 1-*O*-alkyl-2-*O*-acetyl-*sn*-glyceryl-3-phosphocholine) is a potent lipid mediator with a wide variety of biological activities. Studies have demonstrated that PAF is involved in various physiological and pathological events.[1–5] Many cell types are targets for PAF activation. These include human platelets, granulocytes, lymphocytes, endothelial cells and spermatozoa.[1–5] In platelets, PAF interacts with its specific receptor in the membrane initiating a series of biochemical events associated with the signal transduction process.[6]

This study was performed to discern the effect of PAF on the physico-chemical organization of human platelet membranes. The steady-state fluorescence anisotropy of TMA-DPH incorporated into platelet membranes was measured before and after the addition of PAF in the absence or presence of two PAF antagonists, 1-*O*-hexadecyl-2-acetyl-*sn*-glycero-3-phospho(*N,N,N*-trimethyl)hexanolamine.$H_2O$ and (±)-trans-2-(3-methoxy-5-methylsulphonyl-4-propoxyphenyl)-5-(3,4,5-trimethoxyphenyl) tetrahydrofuran (L-659,989). We have also studied the influence of PAF on the fluorescence decay of TMA-DPH which is sensitive to the membrane heterogeneity as previously described.[7]

### Materials and Methods

*Preparation of platelets:* 10 ml of freshly drawn acid–citrate–dextrose anticoagulated blood was centrifuged at 120 × *g* for 15 min at 37°C to prepare platelet rich plasma. Platelets were isolated and washed according to Kubina *et al.*[8] Platelets were finally resuspended in Tyrode's buffer (137 mM NaCl, 2 mM KCl, 12 mM $NaHCO_3$, 0.3 mM $NaH_2PO_4$, 1 mM $MgCl_2$, 2 mM $CaCl_2$, 5 mM glucose) with 0.1% bovine serum albumin and apyrase (Sigma Chemical Co., St Louis, MO). The total platelet count was obtained using a Sysmex E-2500 blood analyser. The platelet suspension was adjusted to a concentration of $4 \times 10^8$/ml. Phase-contrast microscopy (Diaplan, Leica, Milan, Italy) showed that, under the conditions used, control and TMA-DPH platelets remained discoid and functional, being sensitive to thrombin (1 U/ml).[8] Platelets were immediately used for fluorescence measurements. Labelling was carried out in the dark. To a 2.5 ml quartz cuvette containing 2 ml Tyrode's buffer, TMA-DPH was added to give a final concentration of 1 $\mu$mol/l. An aliquot of the platelet suspension was added to give a final concentration of $10^7$ platelets/ml. Platelets were activated by addition of PAF (Sigma Chemical Co., St Louis, MO, USA) at a final concentration of $10^{-7}$ M as previously described.[9] The PAF antagonist, L-659,989 (kindly donated by Dr W. H. Parsons, Merck Sharp & Dohme Research Laboratories, NJ, USA) was added at a final concentration of $10^{-6}$ M.[10] The PAF analogue 1-*O*-hexadecyl-2-acetyl-*sn*-glycero-3-phospho-(*N,N,N*-trimethyl)hexanol-amine.$H_2O$ (Nova-Biochem, Switzerland) was added at a final concentration of $4 \times 10^{-6}$ M.[11]

*Fluorescence studies:* Steady-state fluorescence anisotropy ($r_s$) measurements were performed at 37°C

© 1992 Rapid Communications of Oxford Ltd with a Perkin Elmer Spectrofluorimeter MPF-66, equipped with a Perkin Elmer 7300 Personal Computer for data acquisition and elaboration, using TMA-DPH (Molecular Probes Inc., Eugene, OR, USA) as a hydrophobic fluorescent probe as previously described.[12] The computer program calculated fluorescence anisotropy by using the expression $(I_{\parallel} - I_{\perp} \times g))/(I_{\parallel} + (2I_{\perp} \times g))$ where g is an instrumental correction factor, $I_{\parallel}$ and $I_{\perp}$ are respectively the emission intensities with the polarizers parallel and perpendicular to the direction of the polarized exciting light. Fluorescence lifetime measurements were performed as previously described[13] with a multifrequency phase fluorometer. The instrument was equipped with an ISS-ADC interface for data collection and analysis; the excitation wavelength was set at 325 nm (ultraviolet line of a helium/cadmium laser, Liconix Model 4240 NB). The range of modulation frequencies used for TMA-DPH was 7–130 MHz. Data were accumulated at each modulation frequency until the standard deviations of the phase and modulation values were below 0.1° and 0.002, respectively. The fluorescence was measured through a long-pass filter (type RG 370 from Janos Technology, Townshend VT, USA) which showed negligible luminescence. The experimental data were analysed by a model that assumes a continuous distribution of lifetime values characterized by Lorentzian shape centred at a time C and having a width W. For this analysis the program minimizes the reduced chi-square defined by an equation reported elsewhere.[14] The temperature of the samples was maintained at 37°C with an external bath circulator (Haake F3).

*Statistical methods:* The significance of the data obtained were calculated according to Student's *t*-test.

### Results

The background phospholipid fluorescence of platelets at the used concentration was checked prior to each measurement and was less than 0.5% of the fluorescence when TMA-DPH was added. In our conditions we tested if TMA-DPH in unstimulated and PAF-stimulated platelets is located in the outer monolayer of the plasma membrane by dilution experiments. Immediately after dilution of unstimulated and PAF-stimulated platelets the fluorescence intensity completely disappeared (data not shown), indicating that TMA-DPH was located in the outer monolayer in unstimulated platelets and that it had not crossed the plasma membrane during stimulation with PAF.

In Fig. 1 TMA-DPH fluorescence anisotropy ($r_s$) in platelet membranes before and after PAF addition (final concentration $10^{-7}$ M) is shown.

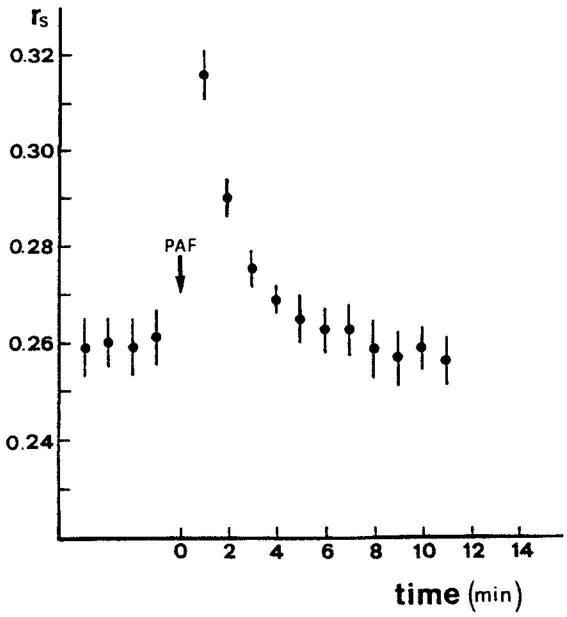

FIG. 1. Steady-state fluorescence anisotropy ($r_s$) of TMA-DPH at 37°C in platelets before and after PAF addition ($10^{-7}$ M). Values are expressed as the mean ± SD of ten samples. The $r_s$ increase after the addition of PAF is significant ($p < 0.01$).

After the addition of PAF a time-limited and significant ($p < 0.01$) increase in the $r_s$ value was observed. After five minutes $r_s$ returned to the baseline value. In the control sample (without PAF) a stable and lasting $r_s$ value was maintained during the measurements. Incubation of PAF, at the concentration used, with TMA-DPH in the absence of platelets, did not produce detectable fluorescence intensity. These data confirm that the observed $r_s$ increase was a consequence of PAF addition to platelets. No changes in fluorescence intensity were evident when PAF was added, indicating that the addition of PAF did not induce platelet aggregation. The absence of aggregation was further checked with phase-contrast microscopy and by single platelet counting.

Figure 2 shows the effect of PAF addition to platelets in the presence of L-659,989. No significant changes ($p > 0.5$) in $r_s$ value were observed after the addition of PAF ($10^{-7}$ M).

Figure 3 shows the effect of PAF addition to platelets in the presence of 1-*O*-hexadecyl-2-acetyl-*sn*-glycero-3-phospho(*N*,*N*,*N*-trimethyl-hexanolamine.$H_2O$. After addition of this PAF antagonist to platelets a significant and stable decrease in $r_s$ value was observed. Consequent addition of PAF ($10^{-7}$ M) induced no significant changes ($p > 0.5$) in $r_s$ value. Control experiments have demonstrated that TMA-DPH labelled platelets, maintained at 37°C for 10 min, showed an increase of $r_s$ after addition of PAF. These data indicate that the effect of PAF on platelets was blocked by the two antagonists. The specificity of the two antagonists was confirmed by activating antagonist-treated

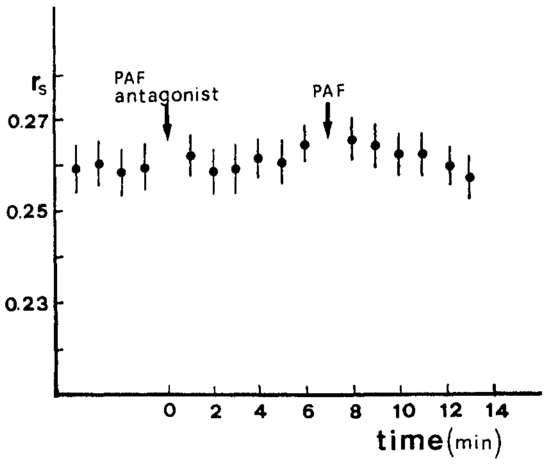

FIG. 2. Steady-state fluorescence anisotropy ($r_s$) of TMA-DPH at 37°C in platelets before and after PAF addition ($10^{-7}$ M) in the presence of PAF antagonist L-659,989 ($10^{-6}$ M). Values are expressed as the mean ± SD of ten samples.

platelets with α-thrombin (0.05 U/ml). Both antagonists showed no inhibition on the increase of $r_s$ induced by thrombin (data not shown).

Figures 4A and 4B show the results of the lifetime distribution analysis of TMA-DPH decay in platelet membranes in the absence and presence of PAF, respectively. In the absence of PAF a two component distribution has been found (Fig. 4A); a long component with an average lifetime value of 5.45 ns and a fractional intensity of 0.78, and a short component with an average lifetime value of 1.24 ns and a fractional intensity of 0.22. The distribution width was 0.18 ns for the long component and 0.09 ns for the short component. The addition of PAF ($10^{-7}$ M) induced a 70% decrease in distribution width of the long component, but no effect on the average lifetime value and fractional intensity was observed. Distribution width, average lifetime, and fractional intensity of the short component were not modified by the addition of PAF.

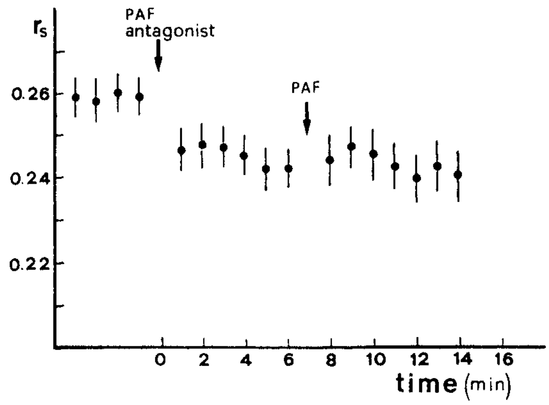

FIG. 3. Steady-state fluorescence anisotropy ($r_s$) of TMA-DPH at 37°C in platelets before and after PAF addition ($10^{-7}$ M) in the presence of PAF analogue 1-*O*-hexadecyl-2-acetyl-*sn*-glycero-3-phospho(*N,N,N*-trimethyl)hexanol-amine.$H_2O$ (4 × $10^{-6}$ M). Values are expressed as the mean ± SD of ten samples.

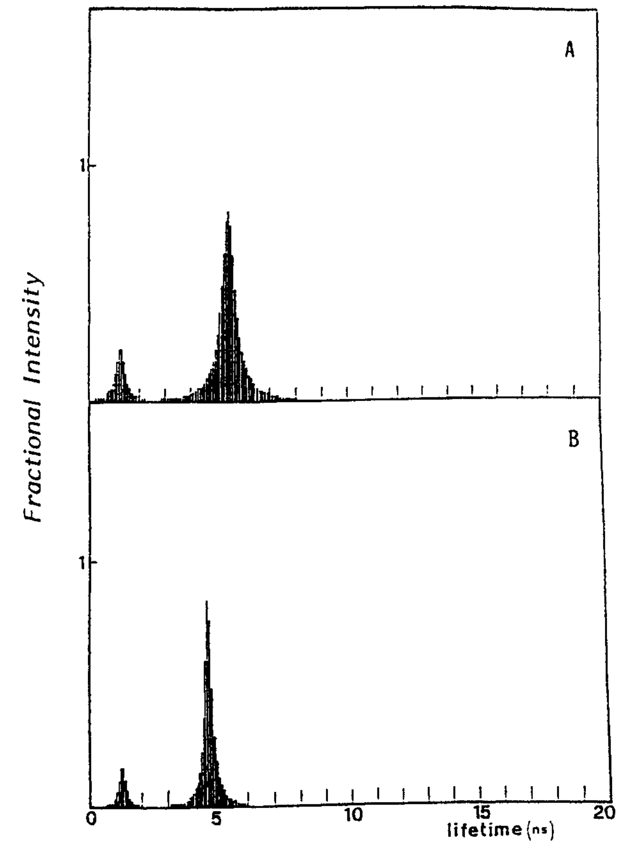

FIG. 4. TMA-DPH lifetime distribution in platelets before (A) and after addition of PAF ($10^{-7}$ M) (B).

### Discussion

Human platelets have both intra- and extracellular specific PAF receptors.[6,15,16] The interaction of PAF with its specific extracellular receptor triggers a complex cascade of biochemical events. These events include the stimulation of GTPase,[17] transient elevation of intracellular $Ca^{2+}$,[18] coupling to both adenylate cyclase and phospholipase C,[6,19] and protein phosphorylation.[20] The response of platelets to PAF is transient and PAF desensitizes its receptor. This phenomenon of time-dependent attenuation of responsiveness is common to many receptor systems.[6,21]

The organization of cell membrane components depends on a number of factors, including the protein and lipid composition of the membrane, receptor occupancies, and activities of membrane proteins.[22] Cell membranes respond to stimuli and integrate the enzymatic responses and functions by rearrangement of membrane configuration. Depending on the nature of the stimulus and the state of the membrane, different biochemical events take place at the membrane level that influence membrane organization. For example, the activation of membrane phospholipases produces rapid changes in membrane phase behaviour, alteration in domains, and inhibition or activation of membrane proteins.[22]

In recent years fluorescent probes, such as DPH and its cationic derivative TMA-DPH, have been used widely in the study of the physico-chemical state of biological membranes.[23,24] Their photophysical properties are affected by the physico-chemical changes of the microenvironment where the probes are located. Because of its hydrophobic structure, TMA-DPH incorporates into the membrane but remains at the lipid–water interface region with its cationic residue.[8] TMA-DPH $r_s$ reflects the packing of membrane lipid fatty acid chains and can be related to the order parameter S, if certain precautions are taken.[25,26] This packing can change upon stimulation of certain cell types.[27,28]

Our results indicate that PAF induces a time-limited increase of lipid order in the exterior part of platelet membranes. In the presence of PAF antagonists the described effect of PAF is totally abolished. The TMA-DPH fluorescence lifetime value is dependent on the dielectric constant of the medium where it is embedded.[29] Therefore the width of the lifetime distribution can be related to the different physico-chemical properties of the environment surrounding the probe. The distribution analysis, although based on phenomenological ground, offers a good description of membrane heterogeneity.[7,13,14] In our distribution analysis it was necessary to include a second component at a shorter lifetime with a very low fractional intensity. The origin of this second component is still debated; for DPH this component has been referred to as a photochemical derivative of the probe[30] or alternatively it can represent a fraction of probe molecules localized in a very polar environment.[31] In any case the relative fractional intensity of this short component did not change significantly after addition of PAF.

Our data for TMA-DPH lifetime distribution show that PAF induced no changes in the central lifetime value, whereas a narrowing in the distribution width of the long component is observed, indicating a decrease in the membrane microheterogeneity. The observed changes in lipid packing and in membrane heterogeneity in activated platelets could result from modifications in lipid metabolism through the activation of endogenous phospholipases.[6,19,20] An alternative explanation could be based on a redistribution of membrane proteins during activation.[32] In any case a transition of the probe to a more homogeneous environment seems to take place during PAF activation.

Our study demonstrates that PAF interaction with platelet membranes induces a time-limited increase in lipid packing of the exterior part of the membrane and a decrease in membrane heterogeneity. Because these changes in the physico-chemical structure of the membrane can be blocked by PAF antagonists, it seems likely that they are attributed to PAF–receptor interaction and the biochemical events associated with signal transduction.

### References

1. Braquet P, Touqui L, Shen TY, Vargaftig BB. Perspectives in Platelet-activating Factor Research. *Pharmacol Rev* 1987; **39:** 97–145.
2. Synder F, Lee TC, Blank ML. Platelet Activating Factor and Related Ether Lipid Mediators. Biological activities, metabolism and regulation. *Ann N Y Acad Sci* 1989; **568;** 35–43.
3. Struck A, Ten Cate JW, Hosford D, Mencia-Huerta J-M, Braquet P. The Synthesis, Catabolism, and Pathophysiological Role of Platelet-activating Factor. *Adv Lipid Res* 1989; **23:** 219–276.
4. Koltai M, Hosford D, Guinot P, Esanu A, Braquet P. Platelet Activating Factor (PAF). A Review of its Effects, Antagonists and Possible Future Clinical Implications (part I). *Drugs* 1991; **42(1):** 9–29.
5. Koltai M, Hosford D, Guinot P, Esanu A, Braquet P. PAF. A Review of its Effects, Antagonists and Possible Future Clinical Implications (part II). *Drugs* 1991; **42(2):** 174–204.
6. Hwang S-B. Specific Receptors of Platelet-activating Factor, Receptor Heterogeneity, and Signal Transduction Mechanisms. *J Lipid Mediators* 1990; **2:** 123–158.
7. Fiorini R, Valentino M, Glaser M, Gratton E, Curatola G. Fluorescence Lifetime Distribution of 1,6-diphenyl-1,3,5-hexatriene Reveals the Effect of Cholesterol on the Microheterogeneity of Erythrocyte Membrane. *Biochim Biophys Acta* 1988; **939:** 485–492.
8. Kubina M, Lanza F, Cazenave J-P, Laustriat G, Kuhry J-G. Parallel Investigation of Exocytosis Kinetics and Membrane Fluidity Changes in Human Platelets with the Fluorescent Probe, Trimethylammonio-diphenylhexatriene. *Biochim Biophys Acta* 1987; **901:** 138–146.
9. Kantar A, Giorgi PL, Curatola G, Fiorini R. Effect of PAF on Erythrocyte Membrane Heterogeneity: A fluorescence study. *Agents and Actions* 1991; **32:** 347–350.
10. Ponpipom MM, Hwang S-B, Doebber TW *et al.* (±)-Trans-2-(3-Methoxy-5-methylsulfonyl-4-propoxyphenyl)-5-(3,4,5-trimethoxyphenyltetrahydrofuran (L-659,989), a Novel, Potent PAF Receptor Antagonist. *Biochem Biophys Res Commun* 1988; **150:** 1213–1220.
11. Tokumura A, Homma H, Hanahan DJ. Structural Analogs of Alkylacetylglycerophosphocholine Inhibitory Behavior in Platelet Activation. *J Biol Chem* 1985; **260:** 12710–12714.
12. Fiorini R, Curatola G, Kantar A, Giorgi PL, Bertoli E, Tanfani F. Steady State Fluorescence Polarization and Fourier Transform Infrared Spectroscopy Studies on Membranes of Functionally Senescent Erythrocytes. *Biochem Inter* 1990; **20:** 715–724.
13. Fiorini R, Valentino M, Gratton E, Bertoli E, Curatola G. Erythrocyte Membrane Heterogeneity Studied using 1,6-Diphenyl-1,3,5-hexatriene Fluorescence Lifetime Distribution. *Biochem Biophys Res Commun* 1987; **147:** 460–466.
14. Fiorini R, Valentino M, Wang S, Glaser M, Gratton E. Fluorescence Lifetime Distributions of 1,6-Diphenyl-1,3,5-hexatriene in Phospholipid Vesicles. *Biochemistry* 1987; **26:** 3864–3870.
15. Braquet P, Godfroid JJ. Conformational Properties of PAF-acether Receptor on Platelets based on Structure-activity Studies. In: Synder F, ed. *Platelet-Activating Factor and Related Lipid Mediators.* New York: Plenum Press, 1987; 191–235.
16. Hwang S-B, Lam MH. Species Difference in the Specific Receptor of Platelet Activating Factor. *Biochem Pharmacol* 1986; **35:** 4511–4518.
17. Hwang S-B. Identification of a Second Putative Receptor of Platelet-activating Factor from Human Polymorphonuclear Leukocytes. *J Biol Chem* 1988; **263:** 3255–3233.
18. Valone FH, Johnson B. Modulation of Cytoplasmic Calcium in Human Platelets by Phospholipid Platelet-activating Factor, 1-O-alkyl-2-acetyl-*sn*-glycero-3-phosphorylcholine. *J Immunol* 1985; **134:** 1120–1124.
19. Crouch MF, Lapetina EG. No Direct Correlation between $Ca^{2+}$ Mobilization and Dissociation of Gi during platelet phospholipase A2 activation. *Biochem Biophys Res Commun* 1988; **153:** 21–30.
20. Lapetina EG, Siegel EL. Shape Change Induced in Human Platelets by Platelet-activating Factor. Correlation with the Formation of Phosphatidic Acid and Phosphorylation of a 40,000-dalton Protein. *J Biol Chem* 1983; **258:** 7241–7245.
21. Henson PM. Activation and Desensitization of Platelets by Platelet-activating Factor (PAF) Derived from IgE-sensitized Basophils 1. Characteristics of the Sensory Response. *J Exp Med* 1976; **143:** 937–952.
22. Kinnunem PKJ. On the Principles of Functional Ordering in Biological Membranes. *Chem Phys Lipids* 1991; **57:** 375–399.
23. Fiorini R, Gratton E, Curatola G. The Effect of Cholesterol on Membrane Microheterogeneity: A study using 1,6-diphenyl-1,3,5-hexatriene fluorescence lifetime distribution. *Biochim Biophys Acta* 1989; **1006:** 198–202.

24. Collins JM, Grogan WM. Comparison of Steady-state Anisotropy of the Plasma Membrane of Living Cells with Different Probes. *Biochim Biophys Acta* 1991; **1067**: 171–176.
25. Van Blitterswijk WJ, Van Hoeven RP, Van Der Meer BW. Lipid Structural Order Parameters (Reciprocal of Fluidity) in Biomembranes Derived from Steady-state Fluorescence Polarization Measurement. *Biochim Biophys Acta* 1981; **730**: 323–332.
26. Pottel H, Van Der Meer W, Herreman W. Correlation between the Order Parameters and the Steady-state Fluorescence Anisotropy of 1,6-Diphenyl-1,3,5-Hexatriene and the Evaluation of Membrane Fluidity. *Biochim Biophys Acta* 1983; **730**: 181–186.
27. Lenaz G, Curatola G, Fiorini R, Parenti-Castelli G. Membrane Fluidity and its Role in the Regulation of Cellular Processes. *Biology of Cancer* 1983; **2**: 25–34.
28. Fiorini R, Curatola G, Bertoli E, Giorgi PL, Kantar A. Changes of Fluorescence Anisotropy in Plasma Membrane of Human Polymorphonuclear Leukocytes during the Respiratory Burst Phenomenon. *FEBS Lett* 1990; **273**: 122–126.
29. Zannoni C, Arcioni A, Cavatorta P. Fluorescence Depolarization in Liquid Crystals and Membrane Bilayers. *Chem Phys Lipids* 1983; **32**: 179–250.
30. Parasassi T, Conti F, Glaser M, Gratton E. Detection of Phospholipid Phase Separation: A multifrequency phase fluorimetry study of 1,6-Diphenyl-1,3,5-Hexatriene fluorescence. *J Biol Chem* 1984; **259**: 14011–14017.
31. Klausner RA, Kleinfeld AM, Hoover RL, Karnovsky M. Lipid Domains in Membranes: Evidence derived from structural perturbations induced by free fatty acids and lifetime heterogeneity analysis. *J Biol Chem* 1980; **255**: 1286–1295.
32. Steiner M, Luscher EF. Fluorescence Changes in Platelet Membranes during Activation. *Biochemistry* 1984; **23**: 247–252.

**Received 8 January 1992;**
**accepted in revised form 2 March 1992**
